# Evaluation of Argos Telemetry Accuracy in the High-Arctic and Implications for the Estimation of Home-Range Size

**DOI:** 10.1371/journal.pone.0141999

**Published:** 2015-11-06

**Authors:** Sylvain Christin, Martin-Hugues St-Laurent, Dominique Berteaux

**Affiliations:** 1 Chaire de recherche du Canada en biodiversité nordique and Center for Northern Studies, Université du Québec à Rimouski, Rimouski, Québec, Canada; 2 Département de Biologie, Chimie et Géographie, Center for Northern Studies and Centre for Forest Research, Université du Québec à Rimouski, Rimouski, Canada; Pacific Northwest National Laboratory, UNITED STATES

## Abstract

Animal tracking through Argos satellite telemetry has enormous potential to test hypotheses in animal behavior, evolutionary ecology, or conservation biology. Yet the applicability of this technique cannot be fully assessed because no clear picture exists as to the conditions influencing the accuracy of Argos locations. Latitude, type of environment, and transmitter movement are among the main candidate factors affecting accuracy. A posteriori data filtering can remove “bad” locations, but again testing is still needed to refine filters. First, we evaluate experimentally the accuracy of Argos locations in a polar terrestrial environment (Nunavut, Canada), with both static and mobile transmitters transported by humans and coupled to GPS transmitters. We report static errors among the lowest published. However, the 68^th^ error percentiles of mobile transmitters were 1.7 to 3.8 times greater than those of static transmitters. Second, we test how different filtering methods influence the quality of Argos location datasets. Accuracy of location datasets was best improved when filtering in locations of the best classes (LC3 and 2), while the Douglas Argos filter and a homemade speed filter yielded similar performance while retaining more locations. All filters effectively reduced the 68^th^ error percentiles. Finally, we assess how location error impacted, at six spatial scales, two common estimators of home-range size (a proxy of animal space use behavior synthetizing movements), the minimum convex polygon and the fixed kernel estimator. Location error led to a sometimes dramatic overestimation of home-range size, especially at very local scales. We conclude that Argos telemetry is appropriate to study medium-size terrestrial animals in polar environments, but recommend that location errors are always measured and evaluated against research hypotheses, and that data are always filtered before analysis. How movement speed of transmitters affects location error needs additional research.

## Introduction

Technological improvements now allow researchers to acquire huge amounts of data describing the geographical location of wild animals, whether these travel on land, in the water, or in the air [1]. This has allowed detailed descriptions and sophisticated hypothesis testing on the movements [2], behavior [3], migration [4], and habitat selection and habitat use [5] of hundreds of animal species. Argos satellite telemetry (http://www.argos-system.org) is one of the major telemetry technologies used in the past 30 years [6, 7]. Created in 1986, the Argos system calculates the location of a Platform Transmitter Terminal (PTT) by using the Doppler effect on transmission frequency between a message sent by the PTT deployed on an animal and an orbiting satellite [8, 9]. The Argos system is widely used because observers can easily retrieve positions from their office in near real-time, a great advantage over all techniques relying on intensive field work (e.g., VHF telemetry) or on the retrieval of data stored in the deployed transmitters (e.g., many Global Positioning System (GPS) transmitters) [1, 7].

One potential limitation of the Argos system, however, lies in its lower accuracy compared to the GPS [10, 11], to the point where the error associated with Argos locations can be too large to allow a detailed interpretation of animal movement [11]. CLS, the company operating the Argos system [9], attributes the position of a PTT to one of seven Location Classes (LC). Attribution is based on the geometrical conditions of the satellite pass at the time of receiving messages, and on the stability of transmitter frequency [9]. When ≥ 4 messages are received, an error estimate can be calculated and the location is assigned a LC based on its estimated error radius. The upper bound for each LC is then 250 m (LC3), 500 m (LC2), 1500 m (LC1), or > 1500 m (LC0). With < 4 messages, auxiliary locations LCA (3 messages) and LCB (1 or 2 messages) can be calculated, but no error estimate is provided [9]. While some studies have suggested that LCA and LC1 errors can sometimes be similar [12, 13], errors associated to LCA and LCB can still reach a few kilometers [7, 14]. Finally, LCZ indicates invalid locations [9]. An important final note is that the bounding values for the LC3 to LC0 are not absolute bounds. According to CLS, locations rather have a 68% probability of being between the bounding values (i.e. in a normal distribution, 68.27% of the values lie within one standard deviation of the mean).

Assessing correctly the accuracy of Argos locations is important because distinguishing biological variability from technological inaccuracy and sampling error is key to test biological hypotheses [15, 16]. For example, location accuracy can strongly affect estimated speed of travel for migrating animals [17]. Behavioral patterns can also be hard to detect if location errors are greater than one order of magnitude of the maximum observed step length of movement [15]. This problem has been approached in two ways, either through quantification of measurement error or through filtering of the locations most likely to be erroneous. The error can be evaluated experimentally by placing PTTs at a known location, be it on the ground [17, 18, 19] or on a moving animal [20–22]. Devices providing both GPS and Argos locations can also help evaluating the error associated to Argos locations by considering the GPS positions as true [11, 23]. Unfortunately, transmitters using both GPS and Argos are often too heavy to be deployed on small animals over the long term [24]. The number and the accuracy of Argos locations can be influenced by latitude, animal's behavior (including movement speed [25]) or number of satellites in the sky [18, 26], while topography and canopy do not seem to seriously impact Argos telemetry performance [27]. This slightly differs from findings obtained with GPS telemetry [28], where accuracy was influenced by canopy closure [29–31], topography [32, 33], fix interval [33], device orientation [34, 35] and model [29, 30], tree height/density [36, 37], and daily phase and season [34].

Filtering of Argos locations can be done in several ways. The easiest approach consists in only keeping locations from the best location classes (usually LC3, LC2 and LC1) [38–40]. However, in most studies, a low proportion of locations is found in these classes, resulting in a drastic reduction of the number of data points available for analyses [12, 41–43]. Moreover, as explained above, some location errors can be much larger than the bounding value specified for a given LC [12, 44]. An alternative approach thus uses destructive filters to remove improbable locations, based for example on movement speed, angle of movement or spatial redundancy between consecutive locations [8, 43, 45, 46]. Still another approach relies on state-space models that use the estimated sampling error as part of the estimation process [11, 16, 47–49]. While more complex to implement, the latter approach does improve methods of investigation on animal behavior [47, 50–52].

The increasing speed at which new miniaturized battery-PTT units are developed creates a strong need to refine our understanding of the accuracy of Argos locations (defined here as the mean distance error from a known true position; not to be confused with precision, i.e. the area within which a given percentage of locations are likely to be found [17]). Animals of small size moving over small areas are indeed much more numerous than those of big size moving over large areas, so that hardware miniaturization opens many new research opportunities. This also increases the importance of assessing correctly the accuracy of Argos locations. In addition, Argos accuracy in terrestrial environments is still relatively undocumented compared to marine environments [25, 53]. Besides, while it was suggested that movement might impact negatively the performance of Argos telemetry [25], to our knowledge this was not quantified experimentally.

Many studies have focused on contexts where signal quality can be seriously degraded because animals spend most of their time underwater [13, 54, 55] or often change their flight altitude [53]. Here we rather focus on ground conditions where signal quality and thus location accuracy should be maximal, and we investigate the extent to which detailed biological questions could be answered in these conditions.

Most filtering methods were created for datasets containing lots of bad quality locations, but their ability to improve good quality datasets has never been tested [7, 11, 51]. Polar regions offer exceptional conditions for Argos telemetry, considering that the Argos satellites have a polar orbit and that satellite coverage increases with latitude [26], resulting in a higher, optimal fix rate obtained every day. These conditions allow for an important proportion of good quality locations [56]. We answered three objectives. First, we evaluated experimentally the error associated to Argos locations in a polar terrestrial environment, with both static and mobile transmitters. Second, we used different filtering methods to test how they improved the quality of Argos locations. Finally, we assessed how location error impacted the estimation of home-range size, since this metric is commonly used to quantify animal space use [57], efficiently synthetizes animal movement [58], and initiates most habitat selection analyses at several scales [59]. Since there are debates as to the pros and cons of various home-range size estimators [60, 61], we assessed the effect of Argos location error on two common estimators, the 95% minimum convex polygon (MCP) and the 95% fixed kernel density estimator.

## Materials and methods

### Study area

We worked during July 2012 in the southern plain of Bylot Island (73°N, 80°O) which is part of Sirmilik National Park, Nunavut, Canada (S1 Fig). The area is characterized by flat lowland and upland plateaus intersected by valleys [62]. The tundra vegetation is composed mostly of low shrubs, grasses, mosses and lichens, with no forest cover.

### Ethics statement

All necessary permits were obtained for the described field work on Bylot Island, which is within Sirmilik National Park of Canada (Parks Canada permit #SIR-2011-8212, amended 7 May 2012).

### Materials

We used twelve collars weighing < 115 g each and bearing Argos Platform Transmitter Terminals (PTT) (Model Kiwisat 203, Sirtrack Ltd, Hawkes Bay, New Zealand). The PTTs had a repetition rate of 60 seconds and transmitted daily between 14:00 and 17:00 GMT. To get reference locations of the Argos PTTs, each of them was randomly coupled to one of three GPS receivers (Garmin GPS76) as explained below under *Static tests*. The inaccuracy associated with GPS locations is negligible compared to that associated with Argos locations (Cargnelutti et al. [63] reported a median error < 10m for GPS units used to track wildlife in open areas), thus we considered the GPS locations as true locations.

### Static tests

Our first objective (part 1) consisted in assessing Argos accuracy on static PTTs (static tests) in three classes of topography defined by the percentage of obstructed sky: 0–33% (hilltop), 33–66% (moderate relief) and 66–100% (incised valley). Two replicates were done for the first two classes and only one for the last, due to the rarity of incised valleys in our study area (S1 Fig). For each test, we attached 3 Argos PTTs on wooden stakes located less than 1 m from each other. Each PTT was located about 30 cm above the ground, with its antenna pointing towards the sky. The PTTs were left at the same spot for 6 days. During the first 3 days, they were restarted daily between 12:00 and 13:00 GMT, allowing them to transmit continuously in the following 24 hours after each start. During the last 3 days, they were not restarted daily and thus emitted only from 14:00 and 17:00 GMT. At each site, one GPS receiver was placed on the ground close to the collars. The GPS receivers were programmed to record and store a location every 30 seconds. They were left for a full day or until the batteries were discharged. The coordinates of the reference location for each test site were calculated as the average coordinates of all GPS locations for this site.

### Mobile tests

Our first objective (part 2) consisted in assessing Argos accuracy on mobile PTTs (mobile tests) during 20 sessions when we walked in the tundra with 3 Argos PTTs and one GPS receiver attached on our backpack. Each mobile session lasted > 4 hours. The Argos PTTs were started at the beginning of each session and the GPS was set to record a location every 20 seconds. The tracks performed on 15 sessions were loops originating and ending at our camp, whereas 5 other tracks reflected one-way trips to camp after we were dropped by a helicopter a few kilometers away. Our average walking speed was 3.4 ± 0.4 km.h^-1^.

To determine a reference location for each Argos location obtained during a mobile test, we looked for the two GPS locations that were obtained just before (GPS1) and after (GPS2) the Argos location, and then calculated through linear interpolation a reference GPS location at the time of the Argos location. The timestamp provided by Argos for a given location is the average time between the first and the last message used to calculate this location (D. Stakem, Service Argos, pers. comm.), and the average duration of an Argos satellite pass is ca. 10 min. We therefore calculated a reference GPS location only when the time difference between GPS1 and GPS2 was <10 minutes, as gaps in our GPS tracks sometimes happened due to poor reception or battery failure.

**Fig 1:**
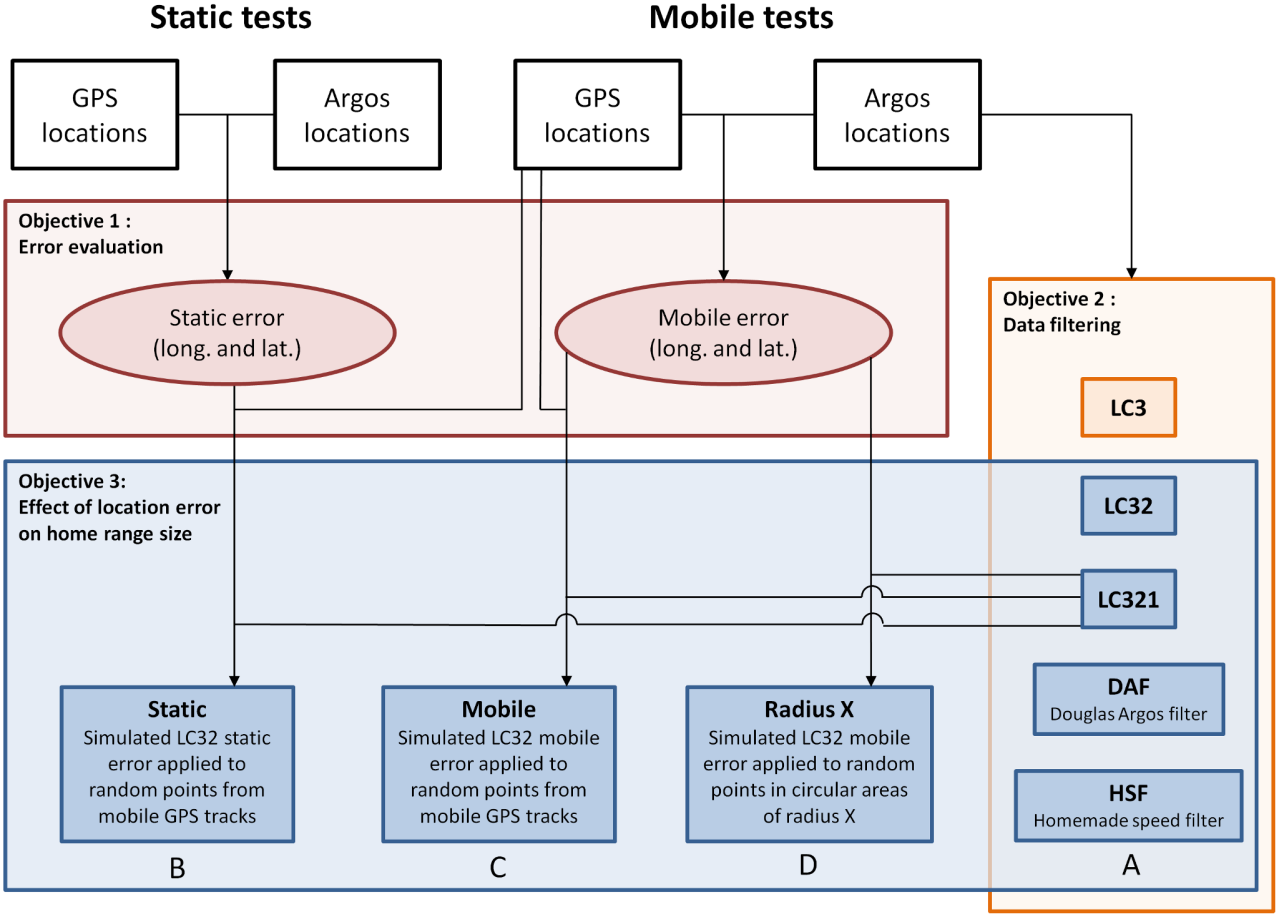
Linkages between research objectives and data structure. White boxes represent raw data collected in the field while conducting tests with static and mobile transmitters. Accuracy of Argos locations (objective 1) was evaluated through comparisons between GPS and Argos locations. The effects of five data filtering procedures on the quality of the resulting datasets were compared during objective 2, using Argos locations obtained during mobile tests as initial dataset. Objective 3 assessed in four ways (A to D) the effect of location error on two estimators of home range size (see methods for details). Note that we did not manage to fully harmonize the logical flow of the study with the reading order of the figure, so that objective 3A appears to the right of 3D. Black lines and arrows identify data sources used to address each objective.

For both static and mobile tests, we defined the error associated to an Argos location as the Euclidean distance between that location and the associated reference GPS location (Fig 1, objective 1).

### Data filtering

To answer our second objective, we filtered our mobile dataset in five ways (Fig 1, objective 2) and assessed each time the resulting error distribution. The first three filters consisted in keeping only the LC3, only the LC3 and LC2 (LC32), and only the LC3, LC2 and LC1 (LC321). The fourth filter was the Douglas Argos filter (hereafter referred to as DAF) [45], which is freely available on the Movebank website (www.movebank.org). Movebank is a free online infrastructure that allows researchers to archive, manage, analyze and share animal tracking data [64], and the DAF is now widely used by wildlife biologists. DAF is a destructive filter that flags implausible locations based on criteria such as spatial redundancy, movement rates and angles. We filtered our data based on distance, angle and rate of travel between location (Filter method = DAR, or Distance Angle Rate) and kept all location classes (keep_lc = G). Since we worked at a local scale, we kept all near-consecutive locations within 2 km (maxredun = 2) and considered that the maximum sustainable speed for a human walking in the tundra was 8 km.h^-1^ (minrate = 8) and that our tracks were circuitous (ratecoef = 5). These conservative parameters maximized the number of locations retained. Fifth, we applied a homemade speed filter (HSF; S1 File) previously developed and used in our lab [40] and were interested in testing its reliability and efficiency in comparison with the DAF. This destructive filter considers a location to be valid only when one of the two following conditions is met: 1- the speed between that location and the previous one is inferior to a cruise speed determined by the user; 2- the speed between that location and the previous one is inferior to a maximum acceleration speed determined by the user and the duration between the two locations is inferior to the maximum time during which the animal can sustain this maximum speed (also determined by the user). When none of these conditions is met, the last of the two consecutive locations is removed. We used as parameters a cruise speed of 6 km.h^-1^, a maximum acceleration speed of 8 km.h^-1^, and a maximum acceleration duration of 20 min.

### Assessment of the effect of location error on home-range size estimation

To answer our third objective, we evaluated how Argos error impacts home-range size estimation using three consecutive approaches. First, we evaluated the impact of data filtering on home-range size (Fig 1, objective 3A). To this end, we calculated home-range size for the 20 mobile sessions using 1- the unfiltered Argos data (that is, Argos data of all location classes; this corresponds to the Argos locations box of the Mobile tests section in Fig 1), 2- only the LC3 and LC2 locations (LC32), 3- only the LC3, LC2 and LC1 locations (LC321), 4- the DAF-filtered datasets, and 5- the HSF-filtered datasets. We did not calculate home-range size using only the LC3 locations because sample sizes were too small during some mobile sessions.

Second, we evaluated the impact of static (Fig 1, objective 3B) and mobile (Fig 1, objective 3C) Argos error on home-range size. To evaluate the impact of static errors, we applied simulated static errors to our mobile tracks. To that end, we generated the error distributions in latitude and longitude for the LC3 and LC2 locations gathered during the static tests. We then applied random Argos errors from these distributions to random locations from each of the 20 GPS tracks generated during our mobile sessions. The number of dummy Argos locations generated per GPS track was the same as the number of LC32 Argos locations obtained in the field, with the same proportion of locations in each location class. To evaluate the impact of mobile errors, we repeated the same procedure except that we applied Argos errors measured for the LC32 locations during the mobile tests.

Third, we investigated how Argos error influences home-range size estimation when locations are randomly distributed within a circular area. This third approach was needed because the home ranges generated by the tracks obtained during our mobile sessions were likely of a much more elongated shape than those of territorial animals. We thus generated artificial random locations uniformly distributed within circles of different radii (250 m, 500 m, 750 m, 1 000 m, 2 500 m and 5 000 m; Fig 1, objective 3D), thereby investigating how Argos error influences home-range size estimations at different spatial scales. We then applied errors from the mobile LC3 and LC2 distributions to each of these artificial random locations. To be consistent with the simulations done in objectives 3B and 3C, and to allow comparisons between objectives 3B-C-D, we performed 20 simulations for each radius (one for each mobile Argos track), and we generated the same number of artificial locations, with the same proportion of locations in each location class, as in the respective mobile sessions of the LC32 dataset. We repeated all simulations (static, mobile and circular areas) 100 times.

### Minimum convex polygon versus fixed kernel

To assess the effect of Argos location error on the 95% minimum convex polygon (MCP) and the 95% fixed kernel density estimators, we compared each estimate of home-range size generated from Argos data to the size of a reference home range. For the filtered datasets and the static and mobile simulations, reference home ranges were generated from the GPS tracks for each session. For the circular area simulations, the reference datasets were the artificial random locations uniformly distributed in circles of varying radius. As there were always more GPS locations than Argos locations, reference home ranges were estimated by randomly selecting in the reference datasets as many points as there were Argos locations. We repeated this process 1000 times for each Argos home-range size estimation. For each iteration and for each method of home-range size estimation (MCP vs. kernel), we calculated the size of the reference and Argos home range, the ratio of the Argos home-range to the reference home-range (ratio Argos to Reference), and the proportion of Argos locations found within the reference home-range.

To ensure that kernel estimates would be comparable across all scenarios, we used a grid with a fixed cell size of 250 m and a fixed smoothing parameter value of 850 m. This smoothing parameter value is the average value estimated for all filtering treatments of the smoothing parameters from the *ad hoc* method for the Argos and reference home range as defined and implemented in the package “adehabitat” for the R software [65]. Kernel areas were estimated as the 95% contours of the utilization distributions.

### Statistical analyses

To evaluate the impact of topography on the error of static locations (objective 1), we used a linear mixed model with the error as the dependant variable, the visibility and the location classes as fixed effects, and PTTs as random effects to account for potential pseudoreplication. We also used linear mixed models to evaluate the impact of the interaction between test type (static or mobile) and LC on the error. PTTs were again included as random effects. All errors were previously log-transformed to meet the normality requirement of the analysis. Filtered data (objective 2) were explored with descriptive statistics only. We used linear mixed models with the mobile sessions as random factor to determine the influence of treatment on home-range size estimation of simulated and filtered mobile data (objective 3).

Unless otherwise indicated, all data are expressed as mean ± standard deviation. We set the significance level at 0.05 for all tests and performed all statistical analyses in R 3.0.2 (R Core Team, 2013). Data are available from the Dryad Digital Repository [66].

## Results

### Static tests

Static tests generated 2,106 Argos locations, with an average of 3.2 ± 1.2 locations per hour per PTT, and 86.2% of these locations being in LC3, LC2 or LC1 (Table 1, Panels A-F in S2 Fig). One LCZ location was removed from the dataset prior to analyses. We estimated the true position of PTTs from 1,331 to 2,917 GPS locations, depending on the site. The measured 68^th^ error percentiles were slightly greater for LC3 and LC2 locations than the values provided by CLS (Table 1), but values for the other location classes fell within the provided range. Contrarily to our expectations, the 68^th^ error percentile in LCA was 33% smaller than the LC1 one. Longitude errors tented to be greater than latitude errors for LC321, but both errors varied greatly within each LC (Table 1). Our linear mixed model showed that visibility had a significant impact on static error (F_(2, 31.72)_ = 16.72, p < 0.001) and that error in each LC was impacted (F_(10, 2086.04)_ = 3.40, p < 0.001). Comparing least squared means in the model showed that while error did not differ significantly between the 0–33% and 33–66% obstructed sky classes (t_(13,5)_ = 1.22, p = 0.24), the 66–100% obstructed sky class had a significantly lower error than the 0–33% (t_(2054)_ = 5.89, p < 0.001) and the 33–66% obstructed sky classes (t_(91.3)_ = 4.51, p < 0.001).The 68^th^ error percentiles were respectively 27% and 30% smaller over all LCs for the 66–100% obstructed sky class compared to the two other classes.

**Table 1:** Comparison between static and mobile errors for all Argos location classes.

	LC	N	Proportion of total	Mean error ± SD	Longitudinal mean error ± SD	Latitudinal mean error ± SD	Median error	Estimated 68^th^ error percentiles	Error percentiles	
			(%)	(m)	(m)	(m)	(m)	(m)	68^th^ (m)	90^th^ (m)
**Static**	3	964	45.8	259 ± 208	171 ± 165	162 ± 166	213	< 250	298	469
	2	622	29.5	456 ± 359	306 ± 301	283 ± 270	357	250 < < 500	517	953
	1	232	11.0	773 ± 607	540 ± 464	471 ± 486	588	500 < < 1500	920	1613
	0	45	2.1	5330 ± 17085	4152 ± 12852	3123 ± 11321	1454	> 1500	2475	8253
	A	126	6.0	640 ± 866	397 ± 483	419 ± 770	397	NA	618	1128
	B	117	5.6	1195 ± 2143	694 ± 1371	831 ± 1723	478	NA	787	2580
Total		2106	100	557 ± 2656	379 ± 1992	348 ± 1771	299		440	978
**Mobile**	3	267	20.94	556 ± 409	369 ± 310	347 ± 353	470	< 250	643	1007
	2	438	34.35	822 ± 726	533 ± 551	525 ± 583	624	250 < < 500	887	1593
	1	327	25.65	2007 ± 1946	1359 ± 1593	1253 ± 1365	1430	500 < < 1500	2124	3933
	0	161	12.63	6270 ± 9402	4601 ± 8328	3757 ± 4805	3845	> 1500	5780	12850
	A	27	2.12	1988 ± 2166	1350 ± 1750	1144 ± 1575	954	NA	2351	4853
	B	55	4.31	5028 ± 16059	3058 ± 12025	3502 ± 10819	1233	NA	2184	8367
Total		1275	100	1964 ± 5189	1350 ± 4191	1224 ± 3146	864		1433	4105

Columns show the number and proportion of locations obtained for each location class (LC), the mean error, the mean longitudinal and latitudinal errors, the median error, the 68^th^ error percentiles as estimated by CLS [9], and the 68^th^ and 90^th^ error percentiles calculated from Argos locations obtained during static (n = 7) and mobile tests (n = 20) from Argos Platform Terminal Transmitters deployed simultaneously with GPS receivers on Bylot Island, Nunavut, Canada in July 2012.

### Mobile tests

Mobile tests generated 1,275 Argos locations, with an average of 64 ± 20 locations obtained per session (Table 1, Panels G-L in S2 Fig). The proportion of locations of LC3, LC2 or LC1 was still large (80.9%), but the proportion of LC3 decreased by half compared to static tests (20.9% vs. 45.8%) while the proportion of LC1 more than doubled (25.6% vs. 11.0%, Table 1). Mobile errors were significantly greater than static errors for all LCs (linear mixed model, F_(1, 411.02)_ = 105.14, p < 0.001 and the interaction between movement and location classes was also significant (F_(5, 3362.83)_ = 3.35, p = 0.005), and the 68^th^ error percentiles were 1.7 to 3.8 times greater than their static counterparts.

### Data filtering

**Fig 2:**
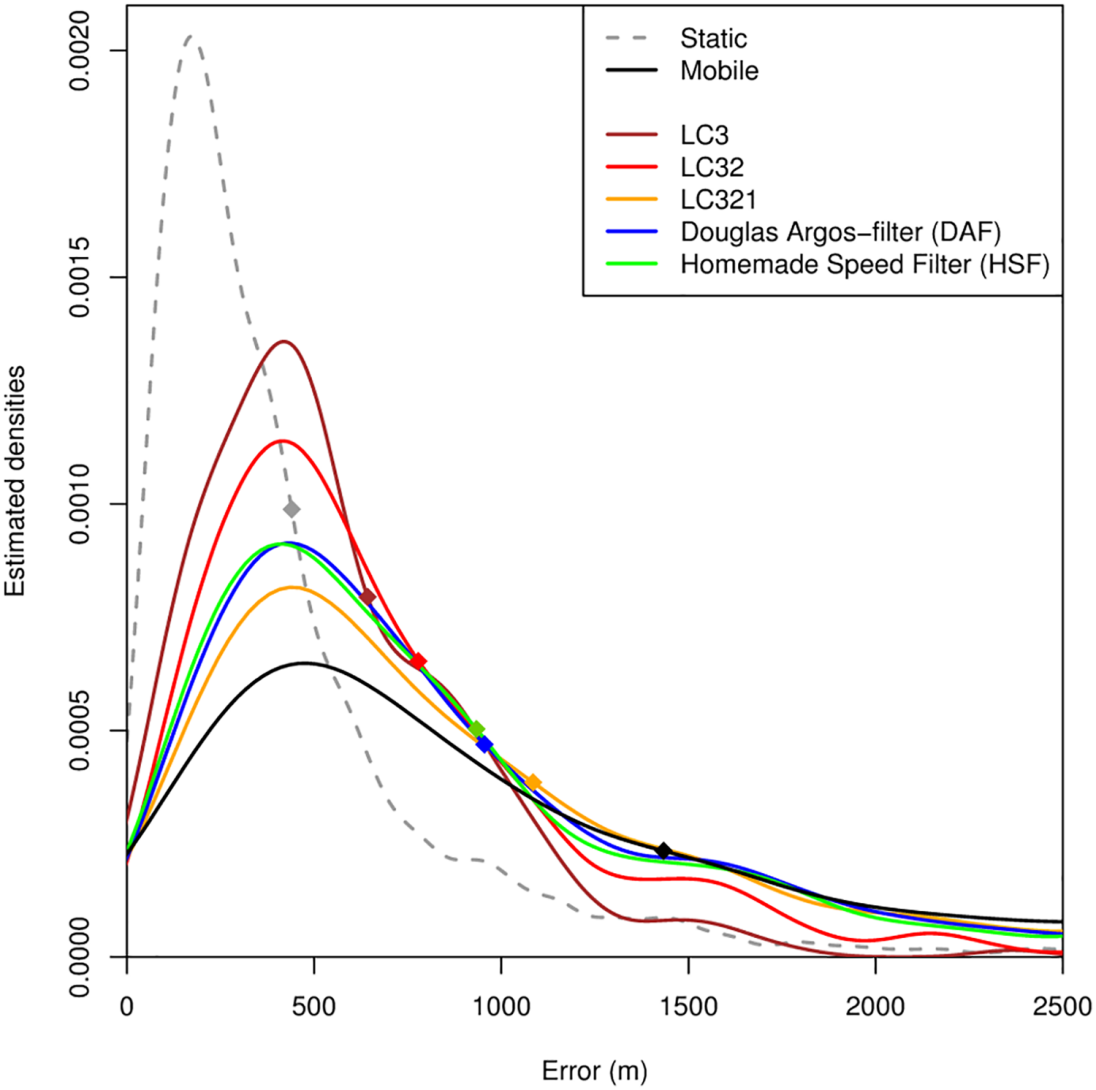
Probability density distributions of the error associated with Argos locations. Locations were obtained during static tests (Static, n = 2,106) and mobile tests (Mobile, n = 1,275). The latter category is decomposed into LC3 locations (n = 267), LC3 + LC2 locations (LC32, n = 705), LC3 + LC2 + LC1 locations (LC321, n = 1,032), locations filtered with the Douglas Argos Filter (DAF, n = 842) and locations filtered with a Homemade Speed Filter (HSF, n = 784). The diamonds indicate the 68th percentile of data. Data were obtained from Argos Platform Terminal Transmitters deployed simultaneously with GPS receivers on Bylot Island, Nunavut, Canada in July 2012.

The DAF and HSF removed respectively 34% and 38.5% of locations, with LC 1, 0 and B being most severely filtered out (Table 2). All filtering methods led to an effective reduction of the 68^th^ error percentiles (Fig 2). The examination of the error distributions shows that the DAF and the HSF performed very similarly. However, keeping only the LC3 or LC32 locations was the most efficient way of reducing location error (Fig 2).

**Table 2:** Comparison of the performance of different filtering methods.

LC	Raw	LC3	LC32	LC321	DAF	HSF
**3**	267	267	267	267	226 (84.6%)	200 (74.9%)
**2**	438	0	438	438	343 (78.3%)	310 (70.8%)
**1**	327	0	0	327	189 (57.8%)	174 (53.2%)
**0**	161	0	0	0	47 (29.2%)	50 (31.1%)
**A**	27	0	0	0	19 (70.4%)	17 (63%)
**B**	55	0	0	0	18 (32.7%)	33 (60%)
**Total**	1275	267 (20.9%)	705 (55.3%)	1032 (80.9%)	842 (66.0%)	784 (61.5%)
**Average no. of loc. per session ± SD**	64 ± 20	13 ± 10	35 ± 19	52 ± 17	42 ± 17	39 ± 15
**Mean error (m ± SD)**	1964 ± 5189	556 ± 409	721 ± 638	1129 ± 1355	895 ± 820	915 ± 964

Columns show the number of Argos locations obtained in each location class (LC), as well as the number of Argos locations retained by the Douglas Argos Filter (DAF) and a Homemade Speed Filter (HSF) applied to data obtained during 20 mobile tests from Argos Platform Terminal Transmitters deployed on Bylot Island, Nunavut, Canada in July 2012. The percentage of locations retained by each filter within each LC is shown in parentheses.

### Assessment of the effect of location error on home-range size estimation

**Fig 3:**
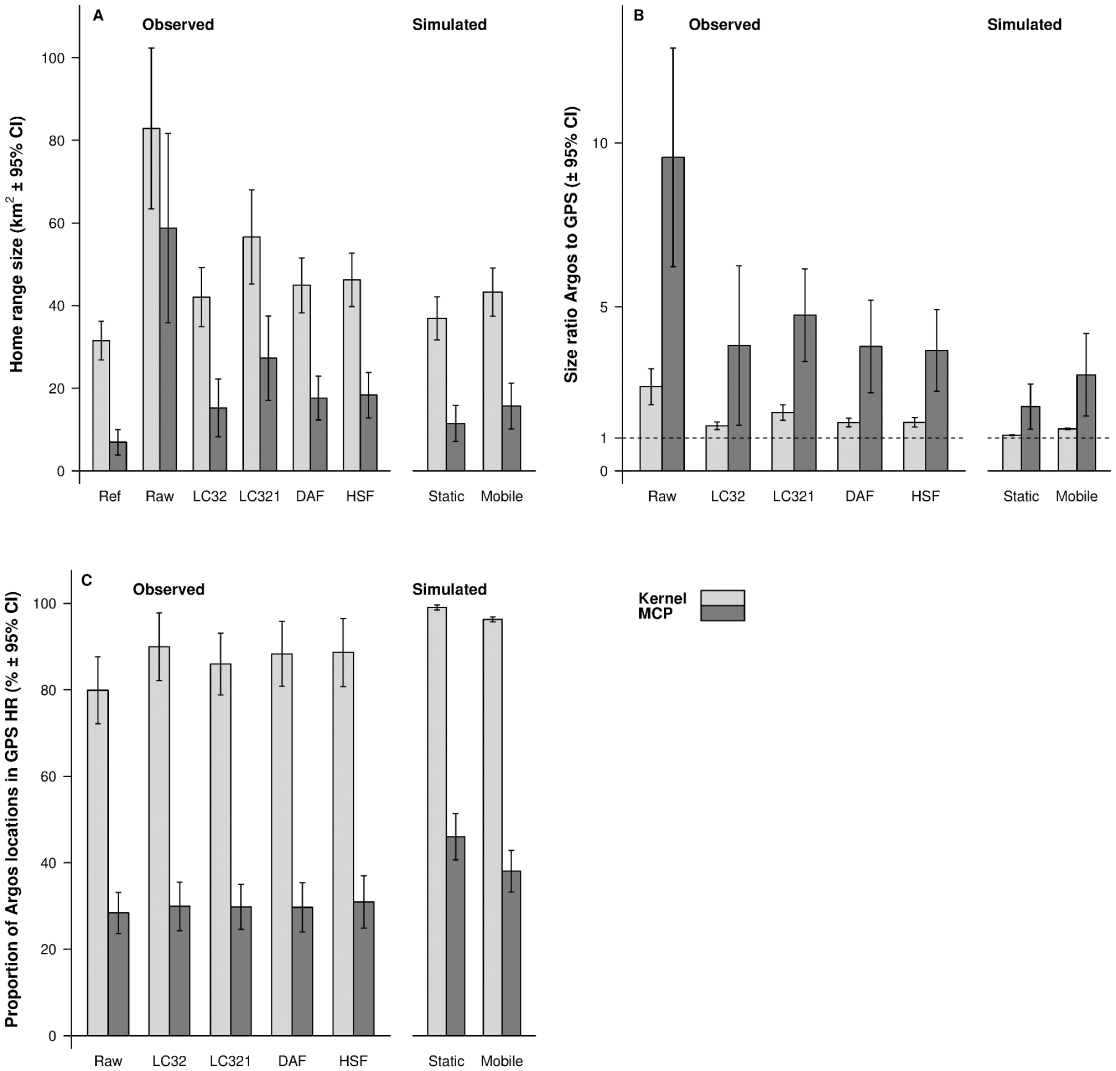
Influence of filtering methods on home-range size estimations. We present (A) the average home-range size, (B) the size ratio Argos to GPS, and (C) the proportion of Argos locations found in GPS home-range size estimates calculated while estimating home ranges based on Argos and GPS locations from mobile experiments using 95% MCP and 95% kernel (h = 850, cell grid size = 250m). The home ranges were estimated for the following scenarios: raw data (Raw), only LC3 and LC2 locations (LC32), LC3 and LC2 and LC1 locations (LC321), data filtered with the Douglas Argos filter (DAF), data filtered with a homemade speed filter (HSF), simulated Argos locations with errors from the static error distribution (Static), and simulated Argos locations with errors from the mobile error distribution (Mobile). Home-range size (A) using the GPS reference area (Ref) is also shown. Data were obtained from Argos Platform Terminal Transmitters deployed simultaneously with GPS receivers on Bylot Island, Nunavut, Canada in July 2012.

Average home-range sizes calculated from GPS locations were 6.9 ± 3.1 km^2^ with the MCP estimator and 31.5 ± 4.6 km^2^ with the kernel estimator (Fig 3A). The four filtering methods resulted in significantly improved estimates of home-range size, as evidenced by the fact that Argos-generated home-range sizes were closer to GPS-generated home-range sizes after the data were filtered (Fig 3A). The lowest size ratios (Fig 3B) and the highest proportions of Argos locations falling in the GPS home range (Fig 3C) were obtained when keeping only locations of LC3 and LC2. Keeping only LC32 indeed decreased home-range size by 74% with the MCP and 49% with the kernel compared to unfiltered data, and the proportion of locations in the reference home range increased by 5% (MCP) and 12% (kernel). Although they did not provide the best results, the Douglas Argos filter and our homemade speed filter led to parameter estimates that did not significantly differ from the LC32 ones (S1 Table).

Size ratios of home ranges and the proportion of locations found in the reference home range calculated for simulated Argos locations with mobile errors were not statistically different from those calculated with the LC32 locations (Fig 3B and 3C, S1 Table), except for the proportion or locations in the reference home range calculated with the MCP (Fig 3C, S1 Table). However, both size ratios and the proportion of locations in reference home ranges calculated for the static dataset differed significantly from the LC32 and mobile datasets, both when using MCP and kernel (S1 Table). As predicted, the static dataset gave consistently estimates of better quality than its counterpart based on mobile error, with mobile home-range size ratios being 49% (MCP) and 18% (kernel) higher than static home-range size ratios, and the proportion of locations in the reference home range being the highest for both methods.

**Fig 4:**
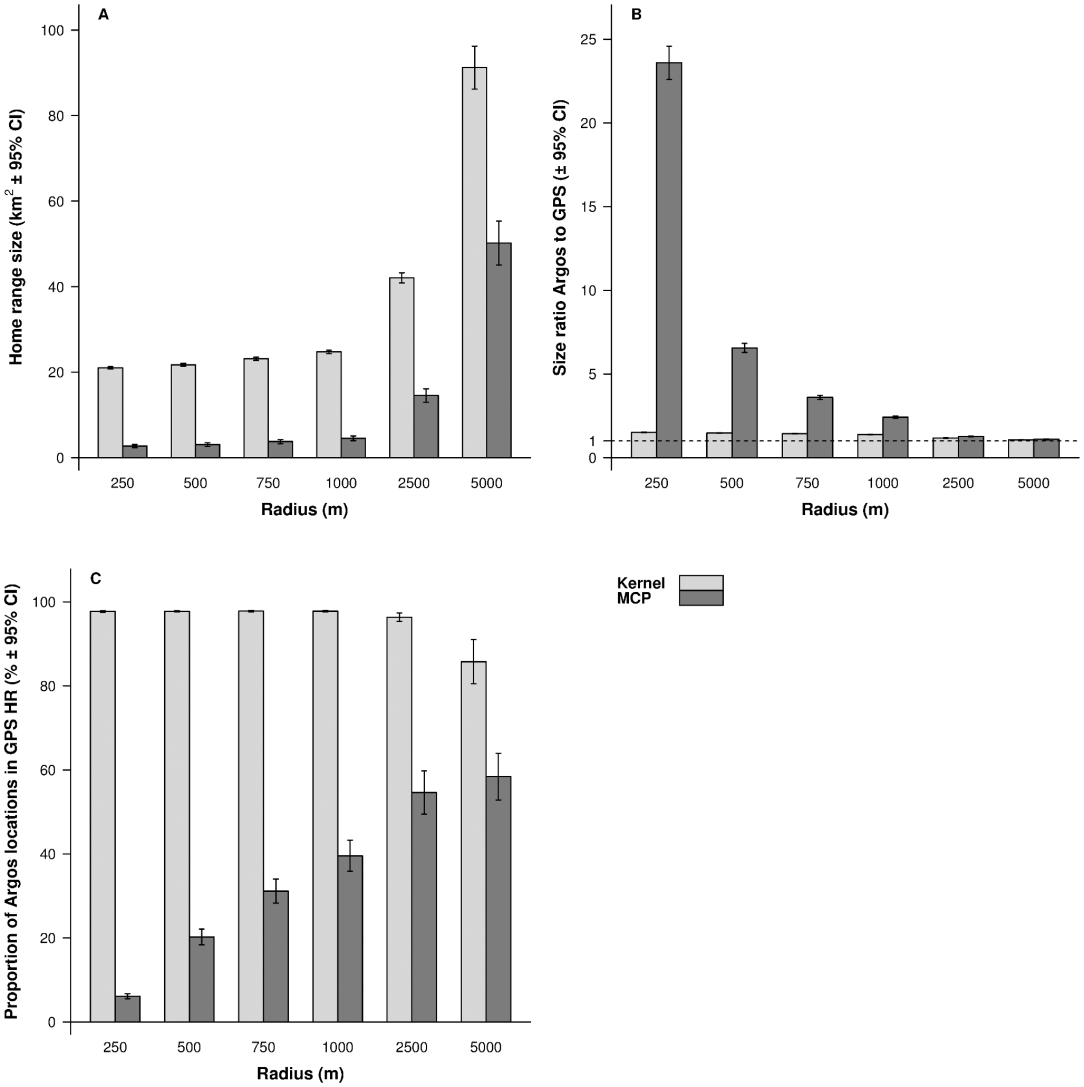
Home-range size estimations for simulated Argos errors in circles of varying radii. We present (A) the average home-range size, (B) the size ratio Argos to GPS, and (C) the proportion of Argos locations found in GPS home-range estimates calculated while estimating Argos and GPS home ranges from mobile experiments using MCP 95% and kernel 95% (h = 850, grid cell size = 250m). Home ranges were estimated for simulated random GPS and Argos locations in circular areas of radius 250 m, 500 m, 750 m, 1,000 m, 2,500 m, and 5,000 m.

The performance of estimations for locations randomly generated in circular areas of various sizes consistently improved as the radius of the circle increased (Fig 4B and 4C). While for a 250-m radius, home-range sizes were overestimated by 2,250% and 50% respectively with MCP and kernel estimators, the overestimations were reduced to 27% and 17% for a 2,500-m radius and to 9% and 6% for a 5,000-m radius. The proportion of locations in the reference home range was ca. 98% with the kernel estimation for radii < 1000 m but started to decrease from radii ≥ 2500 m (Fig 4C). In contrast, this proportion increased steadily with the radius for the MCP estimations.

### Influence of the home-range size estimator on the home-range size estimation

Home-range size estimates were always larger with the kernel estimation method than with the MCP (Figs 3 and 4), especially so for the reference home ranges (Fig 3). The kernel estimator also generated lower size ratios between the reference home ranges and the Argos ones, and generated a higher proportion of locations in the reference home range (Figs 3 and 4).

## Discussion

### Accuracy of Argos locations during static tests

Our static experiments yielded high quality data containing a very high proportion of accurate locations (LC3). To our knowledge, only Sauder et al. [27], working in the northwest of the United States, reported a location set of similar accuracy. Topographic ruggedness improved the accuracy in each location class, a phenomenon that had already been observed [27]. This gain might reflect the screening out of bad quality locations generated when satellites were at a low angle above the horizon [26]. Sauder et al. [27] attributed the high quality of their datasets to recent improvements in satellite technology, PTT technology, and data processing algorithms. We further suggest that the geographical area where PTTs are active might influence the accuracy of reported locations. Indeed, Boyd and Brightsmith [24] recently obtained locations of poor accuracy in Peru, even when using the Kalman filter, introduced in 2011 by CLS. Increased satellite coverage at high latitudes might thus have a strong influence on Argos performance [26]. Other factors, such as PTT power or electromagnetic interference, might also affect Argos location quality differentially according to where the study is conducted [17, 21, 53]. We observed a tendency for a greater error in longitude than in latitude (likely because of the polar orbit of satellites), in good agreement with findings from other studies [23, 24].

### Influence of PTT movement on Argos location error

More than 80% of the locations of our moving PTTs were in LC3, LC2 or LC1, which contrasts with previous reports from Vincent et al. [12] (29,8%), Britten et al. [20] (11%), and Hazel [14] (12.5%). Yet movement did strongly affect the accuracy of Argos locations, since error estimates were almost twice as large in mobile tests than in static tests, whatever the location class. This occurred even though our movement speed (a human pace in the tundra) was relatively low, and confirms that PTT movement is one of the dominant sources of inaccuracy for Argos telemetry [25].

Our simulations showed that the size ratios of home ranges were at least 20% higher for mobile than for static errors. This suggests that a static evaluation of Argos error is not sufficient to assess the accuracy of Argos locations, as is sometimes suggested in the literature [67]. We recommend always evaluating Argos accuracy in conditions similar to those encountered by PTTs deployed on animals, for example through the use of captive animals [12] or through a mix of Argos and GPS technology [23].

### Effect of data filtering on the quality of Argos datasets

The lowest home-range sizes and the highest proportions of locations in reference home ranges were obtained when retaining only high quality location classes (LC3 and LC2), not when applying one of our data filters. This is not surprising, since we only tested destructive filters, which are meant to remove the less accurate locations. Moreover, destructive filters usually remove few good quality locations, mostly by screening out locations from LC0, A and B [46, 55]. However, keeping only locations from LC3 and LC2 resulted in a more reduced dataset than when applying other filtering methods. Keeping only LC3 and LC2 locations might also bias the evaluation of animal space use in favor of some specific areas, such as those where animals rest and thus do not move [39]. In addition, since the error estimates provided by CLS represent only the 68^th^ percentile of the error, some locations can still have an important associated error, even in LC3 [8]. Since no significant difference was found between home-range size estimations based on the LC32 data, Douglas Argos-filter and homemade filter datasets, we suggest that filtering full datasets is more appropriate than using only LC32 locations, whenever possible.

We only tested the efficiency of destructive filters in this study, so the filtered datasets could only be as accurate as the best quality locations. It would be interesting to see how more complex approaches, like state-space models, might improve even more data quality and home-range size estimates [23, 47].

### Influence of home-range delineation method on home-range size estimation

Kernel home ranges had consistently lower size ratios and a higher proportion of locations in the reference home range than MCPs. However, home-range size estimates were always greater with the kernel estimator, which might seem counter-intuitive since the MCP method is known to overestimate home-range size [61]. Our choice of smoothing factor might explain this result, since we used for all our home-range size estimations the average value of the smoothing parameters estimated for all filtering treatments and the reference datasets. By including datasets that contained Argos errors when choosing our smoothing parameter, we might have obtained a larger value than if we only used the reference datasets, and that might have resulted in the overestimation of our reference home ranges [68, 69]. Moreover, we used the *ad hoc* method defined in the adehabitat R package [65] to select our smoothing parameter, which might also lead to bandwidth overestimation [68]. This could explain why the size ratios were lower and why the reference home ranges included a more important proportion of locations. Spatial scale did not influence greatly our kernel results, even though the proportion of locations found in home ranges started to decrease in circles with a 5000-m radius, which might be the result of the smoothing factor becoming too small, thus resulting in an underestimation of the reference home ranges [69]. On the contrary, the MCP method depended much more on the spatial scale, with only a very low proportion of locations found in reference home ranges and a very high size ratio with a 250-m radius. This is not surprising considering that the MCP is sensitive to outliers and is thus more prone to overestimation if the error is important compared to the spatial scale [70]. The size ratios decreased and the proportion of locations in reference home ranges increased as the spatial scale increased, reflecting that, as the spatial scale becomes sufficiently large compared to the measurement error, the error becomes diluted and biological signals can be detected [15]. In the end, even though a better estimation of the kernel bandwidth might be preferable, the choice of the home-range size estimator did not influence our decision when comparing the efficiency of the filters, as both methods yielded comparable results.

## Conclusion

Our methodological study, performed in a terrestrial polar region, yielded a high number of good quality locations with error estimates close to what CLS is advertising. However, PTT movement greatly impacted location accuracy, even at low speeds, and a next step should be to quantify how PTT speed influences accuracy of Argos locations. Data filtering and the assessment of location accuracy in a mobile setup should be two prerequisites before analyzing any Argos dataset in a biological context. We showed that applying the Douglas-Argos filter or a basic speed filter provide similar home range size estimates than keeping only locations falling in the LC3 and LC2 Argos location classes, while retaining more locations, and should therefore be advocated. It is noteworthy that Argos inaccuracy will always lead to an overestimation of home-range size. The importance of this overestimation will be larger for small home ranges. Yet our results suggest that, considering the quality of the data we obtained, Argos telemetry has a high benefit/cost ratio when studying medium-size terrestrial animals in polar environments.

## Supporting Information

S1 Fig Maps showing the location of the study area (top-left) and its general topography (top-right). The two enlarged areas (bottom panels) show the 5 sites used for static tests in three classes of topography (hilltop, moderate relief, incised valley) as well as 20 trips (15 loops + 5 one-way trips) used for mobile tests.
(PNG)

S2 Fig GPS and Argos locations obtained for 6 representative static tests (2 tests for each class of topography; Panels A-F) and 6 representative mobile tests (4 loops and 2 one-way trip; Panels G-L) used to evaluate Argos telemetry accuracy in the High Arctic. Panel titles identify each test and indicate, for static tests, the percentage of Argos locations not seen because they fall out of the graph boundaries. Raw data are available at http://dx.doi.org/10.5061/dryad.bt72k for all 15 static tests (2 sites with 3 PTTs replicated once + 1 site with 3 PTTS without replication) and all 60 mobile tests (15 loops with 3 PTTs and 5 one-way trips with 3 PTTs) performed in this study.
(PDF)

S1 File R script of the homemade speed filter. (TXT)

S1 Table Differences of least squared means between the fixed factors of mixed models. (DOCX)
