# Supplementary figures and images for: Evaluation of Argos Telemetry Accuracy in the High-Arctic and Implications for the Estimation of Home-Range Size
Source: PLoS One. 2015 Nov 6;10(11):e0141999. doi: 10.1371/journal.pone.0141999 (PMC4636246; doi:10.1371/journal.pone.0141999)

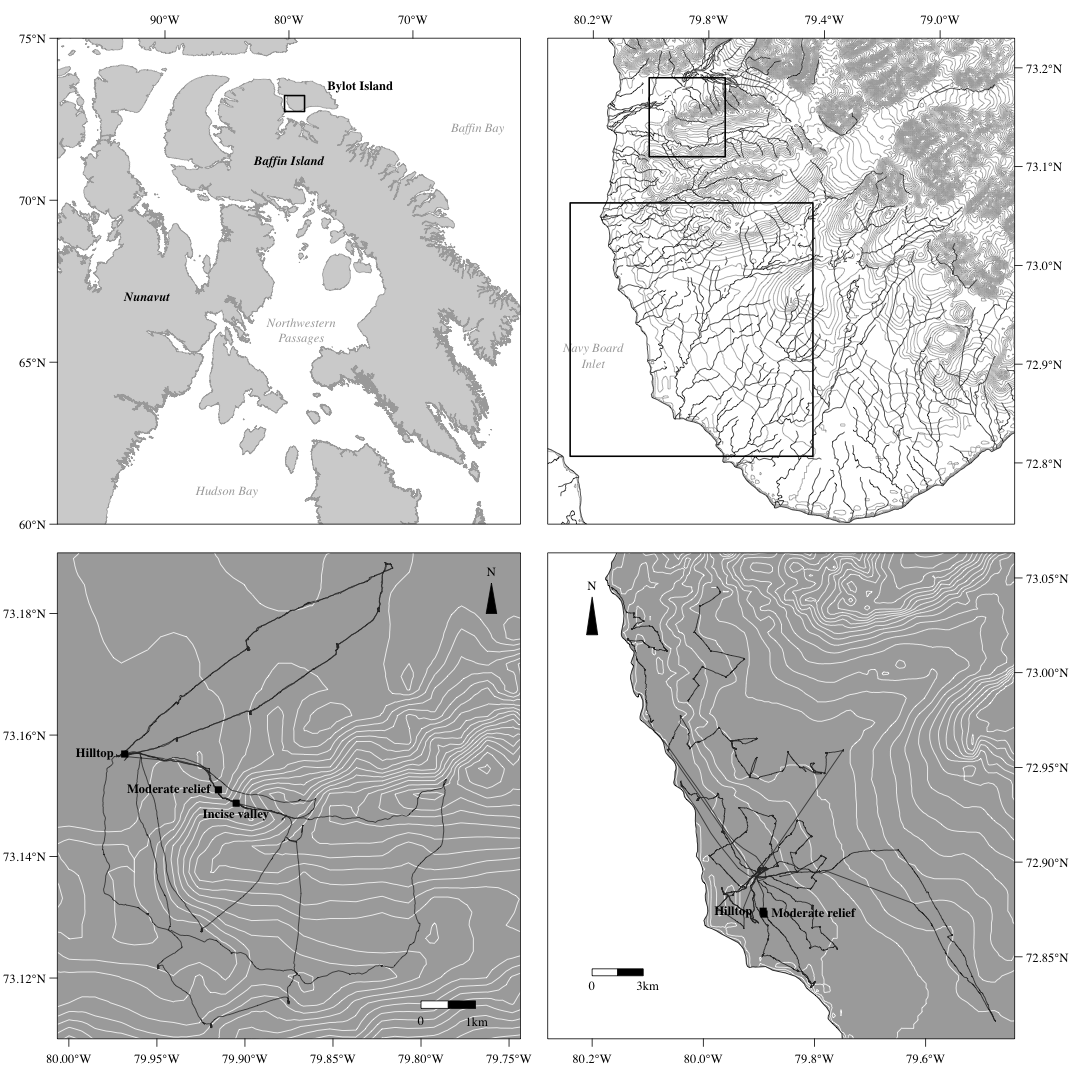

Supplement: S1 Fig — The two enlarged areas (bottom panels) show the 5 sites used for static tests in three classes of topography (hilltop, moderate relief, incised valley) as well as 20 trips (15 loops + 5 one-way trips) used for mobile tests. (PNG) [file pone.0141999.s001.png]
