# Supplementary material for: Evaluation of Argos Telemetry Accuracy in the High-Arctic and Implications for the Estimation of Home-Range Size
Source: PLoS One. 2015 Nov 6;10(11):e0141999. doi: 10.1371/journal.pone.0141999 (PMC4636246; doi:10.1371/journal.pone.0141999)
Supplement: S2 Fig — Panel titles identify each test and indicate, for static tests, the percentage of Argos locations not seen because they fall out of the graph boundaries. Raw data are available at http://dx.doi.org/10.5061/dryad.bt72k for all 15 static tests (2 sites with 3 PTTs replicated once + 1 site with 3 PTTS without replication) and all 60 mobile tests (15 loops with 3 PTTs and 5 one-way trips with 3 PTTs) performed in this study. (PDF) [file pone.0141999.s002.pdf]

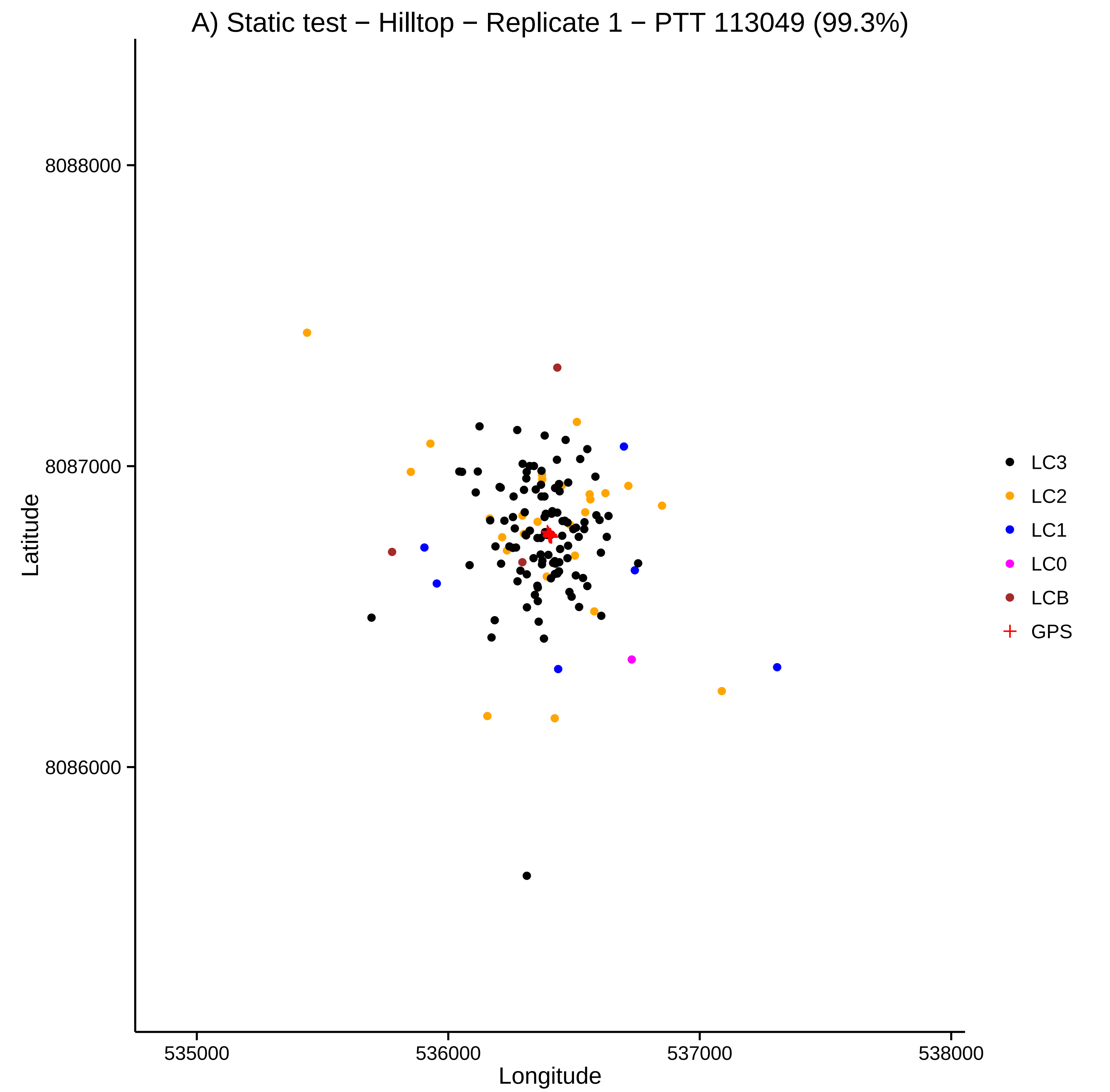
A) Static test − Hilltop − Replicate 1 − PTT 113049 (99.3%)
8088000
8087000
8086000
Latitude
535000
536000
537000
538000
Longitude
LC3
LC2
LC1
LC0
LCB
GPS

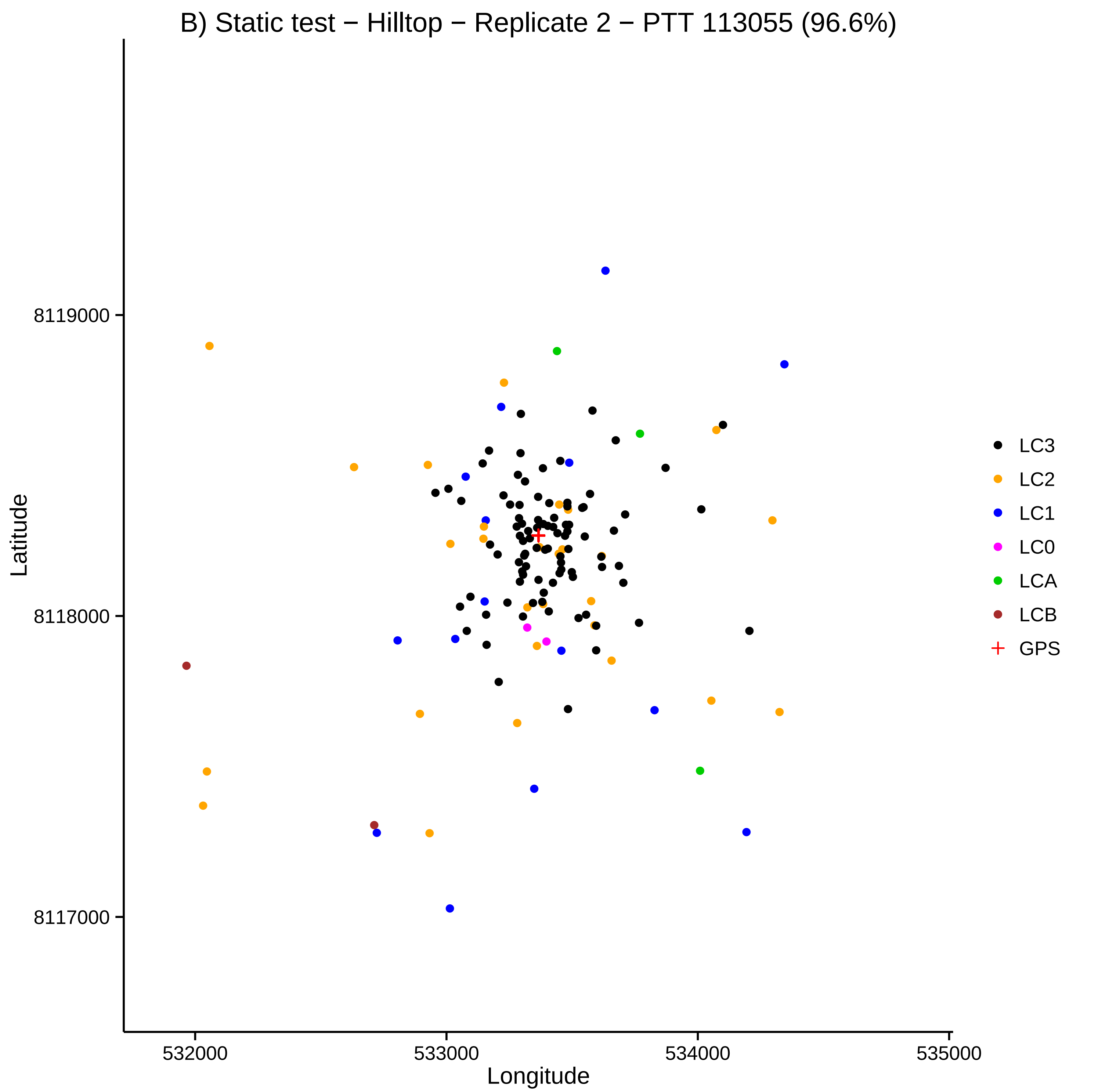
B) Static test − Hilltop − Replicate 2 − PTT 113055 (96.6%)
Latitude
8119000
8118000
8117000
532000
533000
534000
535000
Longitude
LC3
LC2
LC1
LC0
LCA
LCB
GPS

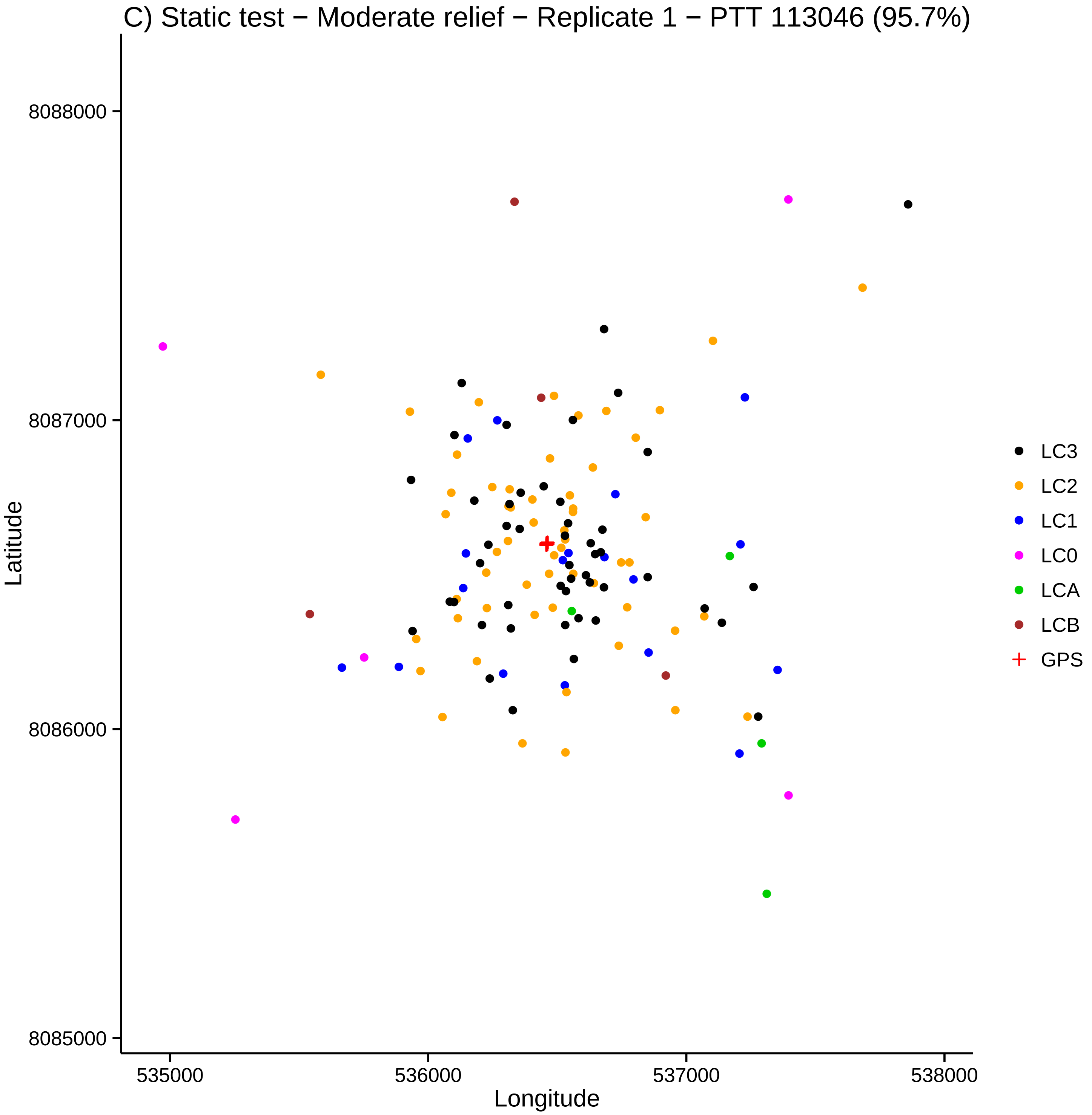

C) Static test − Moderate relief − Replicate 1 − PTT 113046 (95.7%)
Latitude
Longitude
8088000
8087000
8086000
8085000
535000
536000
537000
538000
LC3
LC2
LC1
LC0
LCA
LCB
GPS

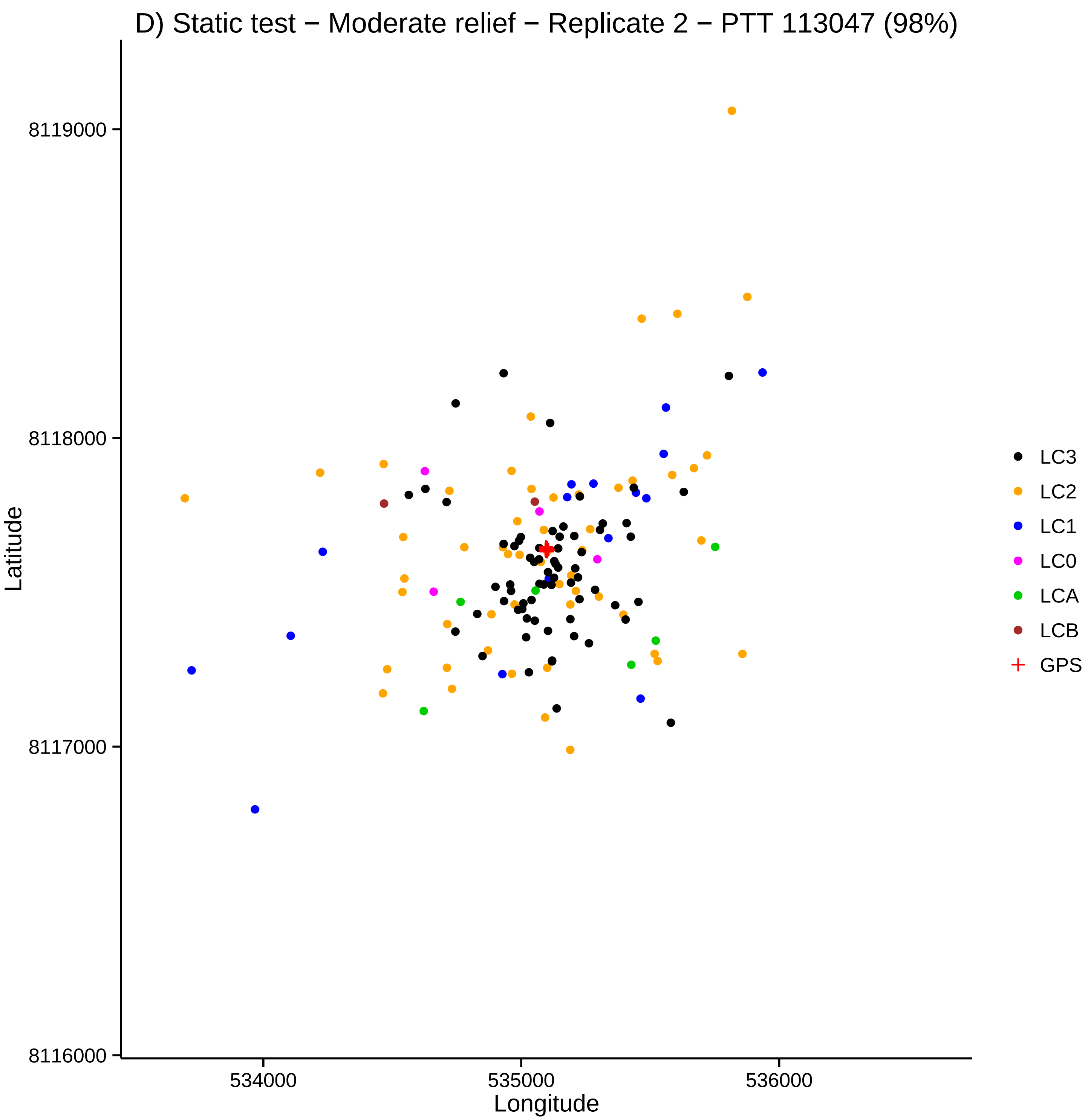
D) Static test − Moderate relief − Replicate 2 − PTT 113047 (98%)
8119000
8118000
8117000
8116000
Latitude
534000
535000
536000
Longitude
LC3
LC2
LC1
LC0
LCA
LCB
GPS

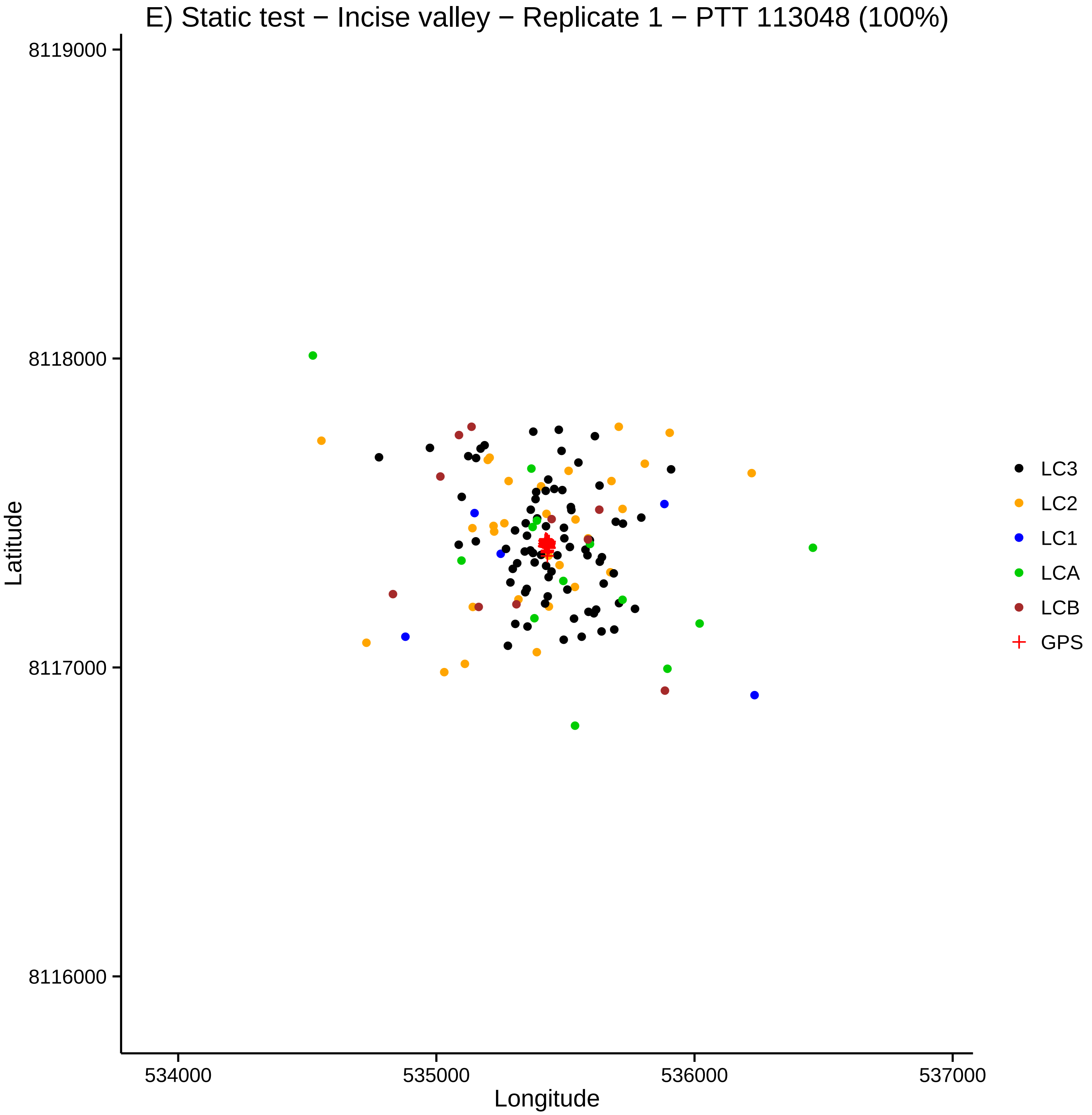

E) Static test − Incise valley − Replicate 1 − PTT 113048 (100%)
8119000
8118000
8117000
8116000
Latitude
534000
535000
536000
537000
Longitude
LC3
LC2
LC1
LCA
LCB
GPS

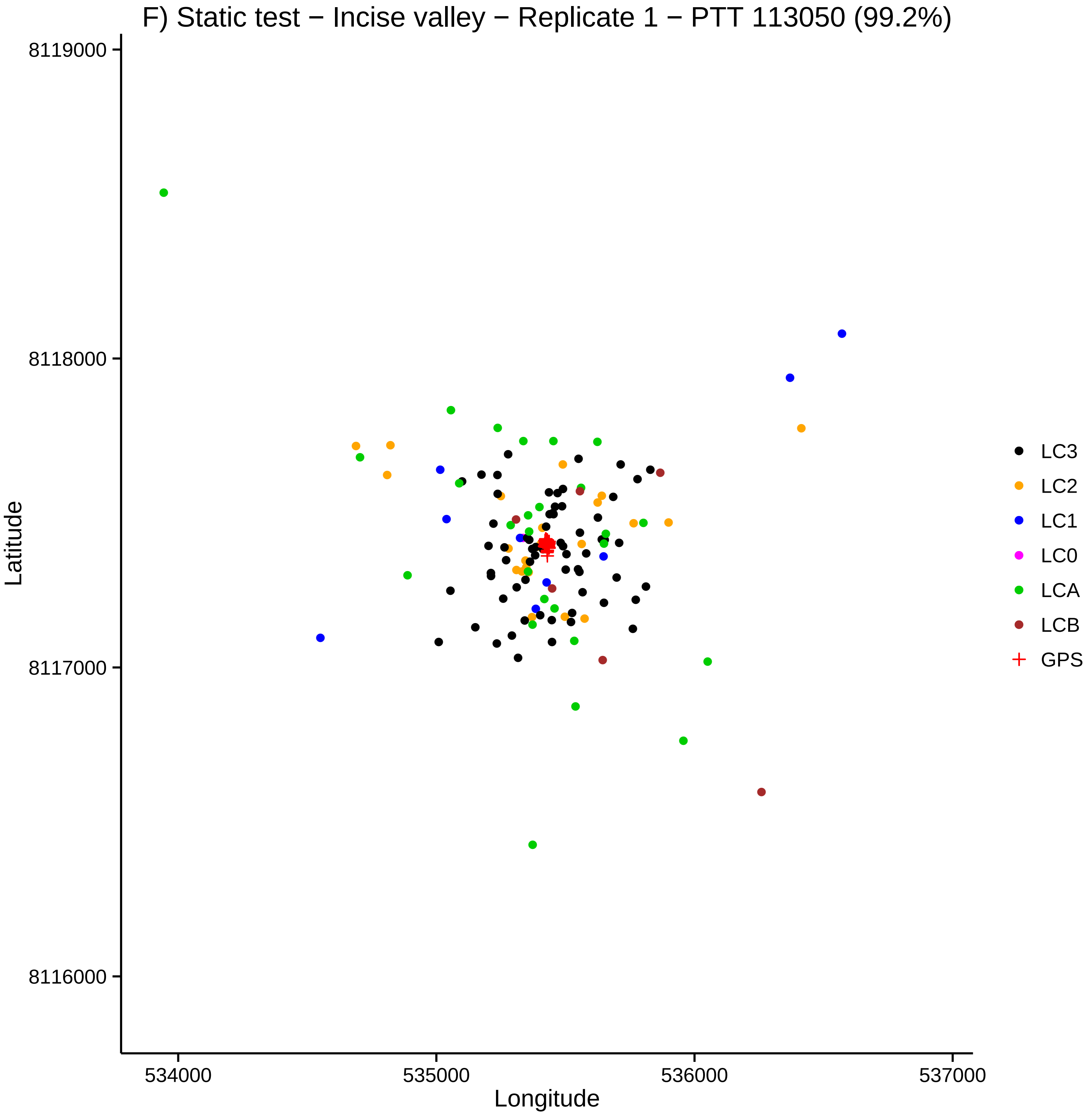

F) Static test − Incise valley − Replicate 1 − PTT 113050 (99.2%)
8119000
8118000
8117000
8116000
Latitude
534000
535000
536000
537000
Longitude
LC3
LC2
LC1
LC0
LCA
LCB
GPS

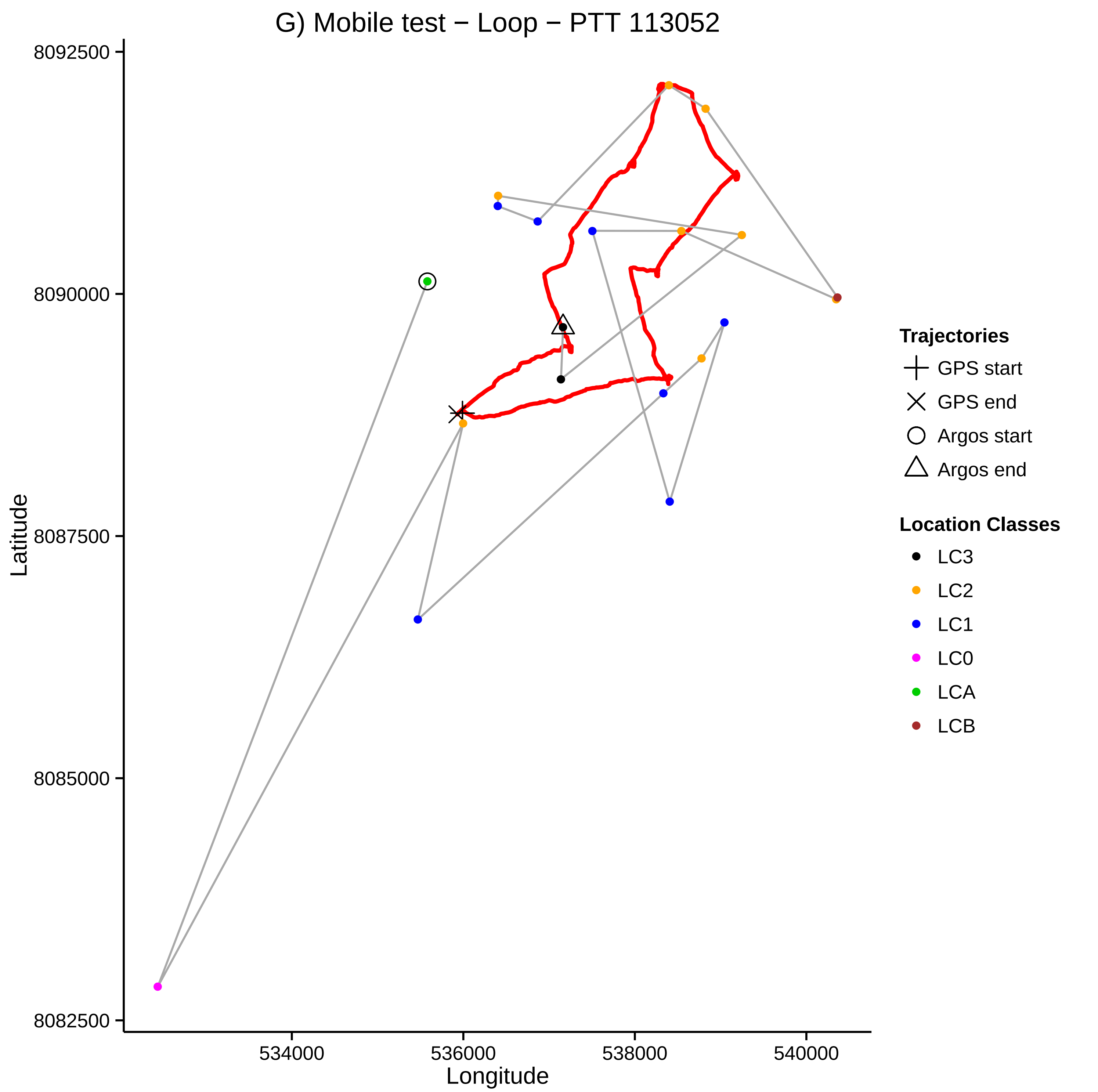

G) Mobile test − Loop − PTT 113052
8092500
8090000
8087500
8085000
8082500
Latitude
534000
536000
538000
540000
Longitude
Trajectories
GPS start
GPS end
Argos start
Argos end
Location Classes
LC3
LC2
LC1
LC0
LCA
LCB

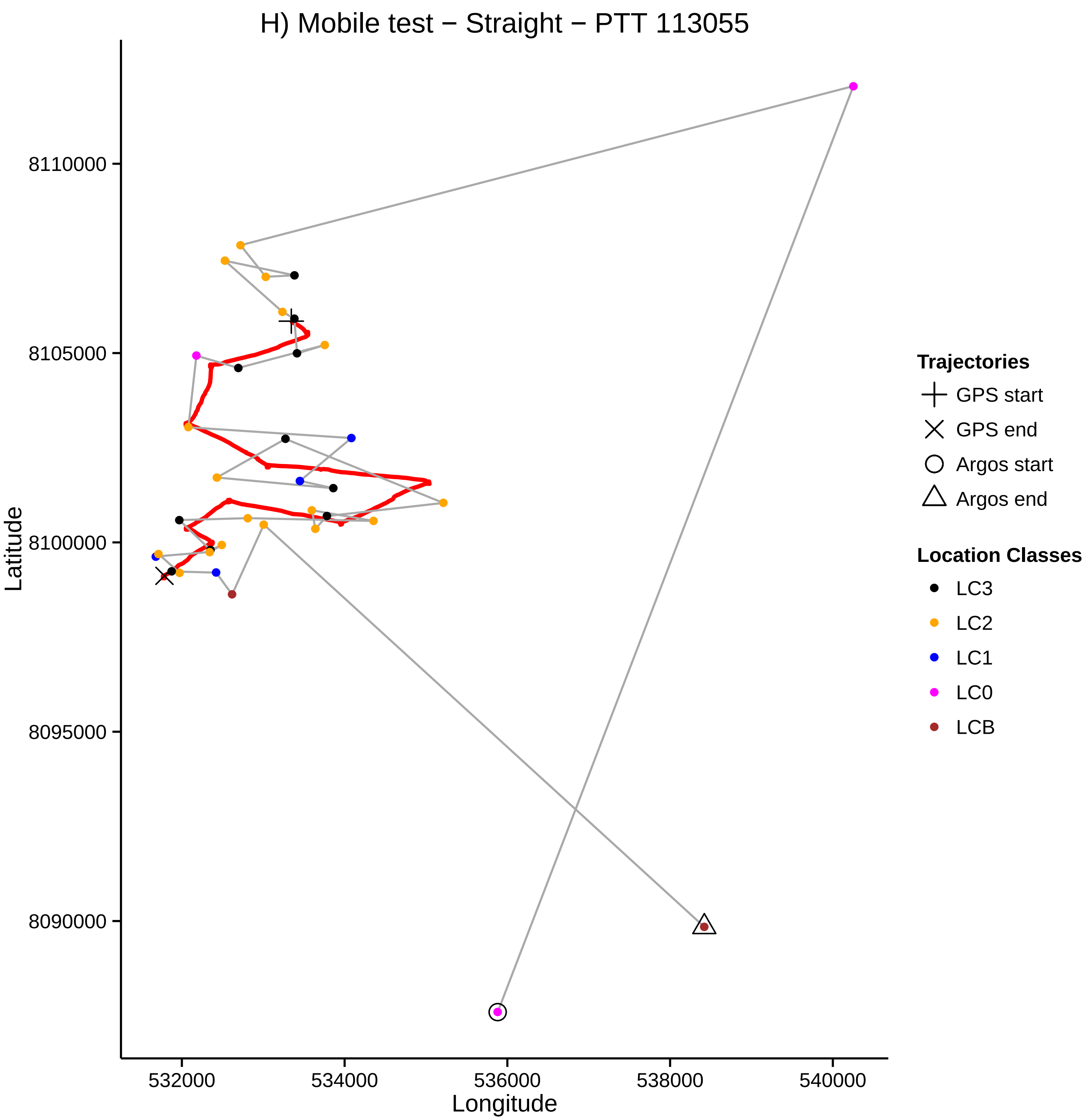

H) Mobile test − Straight − PTT 113055
Latitude
8110000
8105000
8100000
8095000
8090000
Longitude
532000
534000
536000
538000
540000
Trajectories
GPS start
GPS end
Argos start
Argos end
Location Classes
LC3
LC2
LC1
LC0
LCB

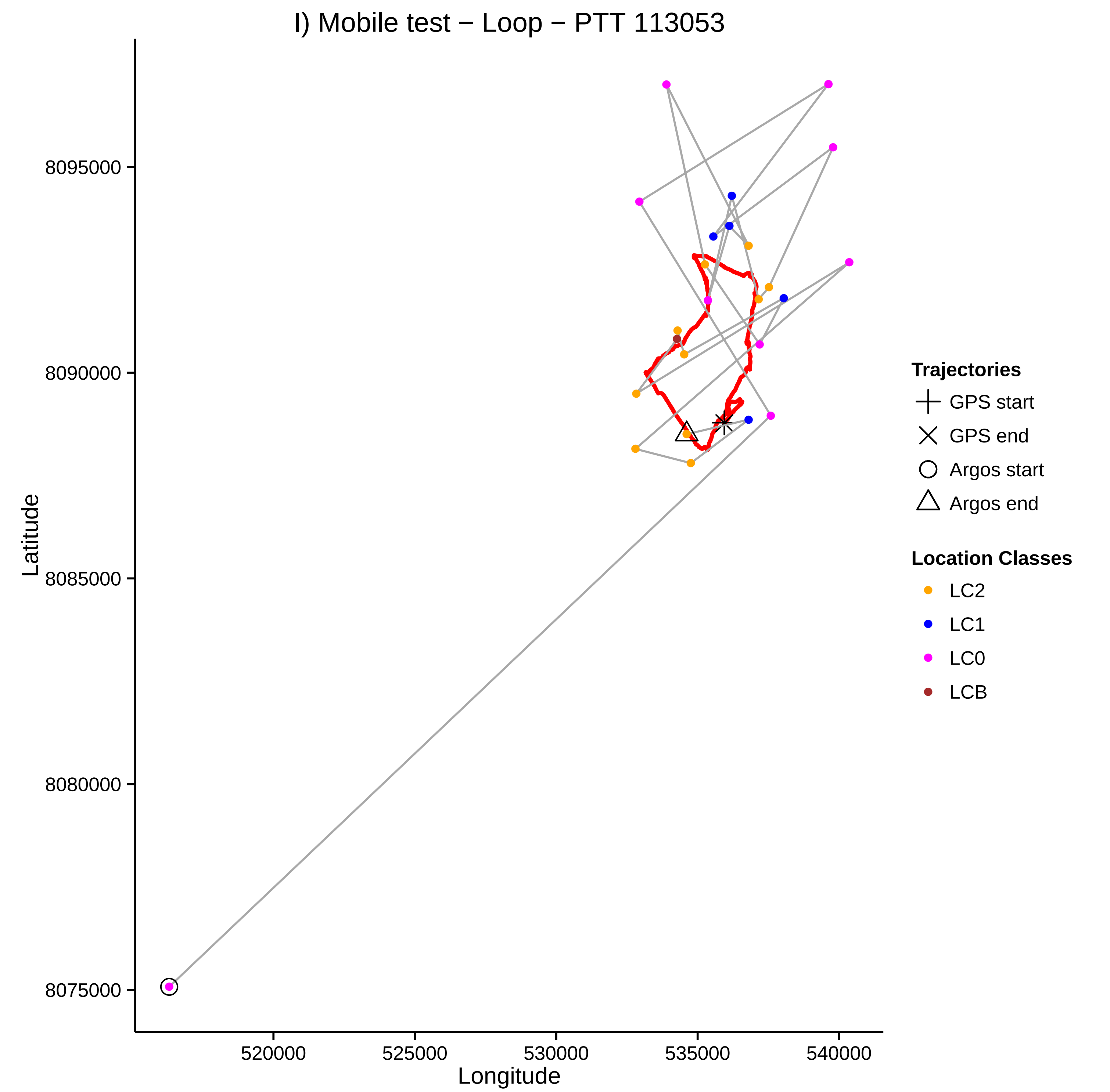

I) Mobile test − Loop − PTT 113053
Latitude
8095000
8090000
8085000
8080000
8075000
520000
525000
530000
535000
540000
Longitude
Trajectories
GPS start
GPS end
Argos start
Argos end
Location Classes
LC2
LC1
LC0
LCB

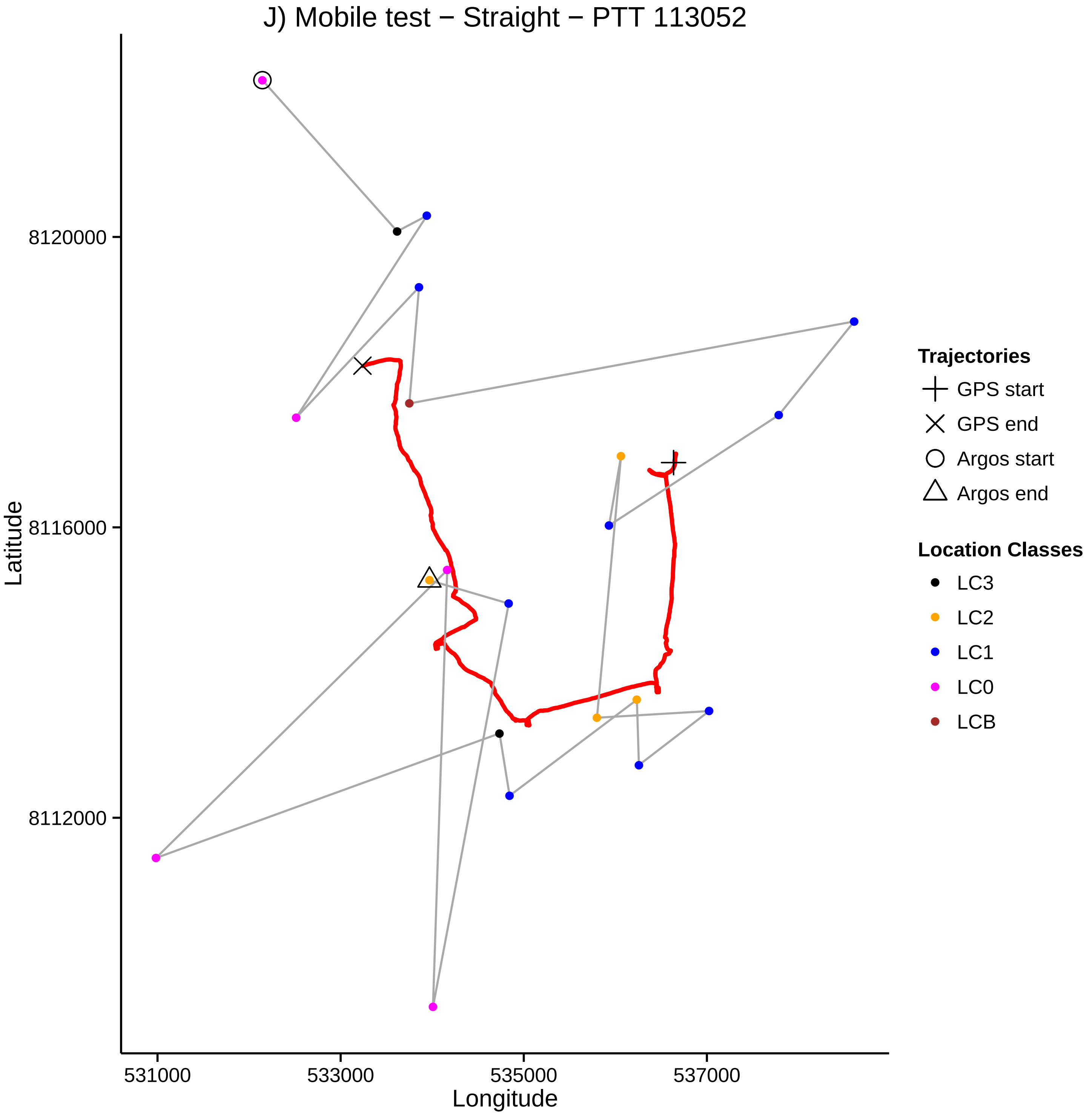
J) Mobile test − Straight − PTT 113052
Latitude
8120000
8116000
8112000
531000
533000
535000
537000
Longitude
Trajectories
GPS start
GPS end
Argos start
Argos end
Location Classes
LC3
LC2
LC1
LC0
LCB

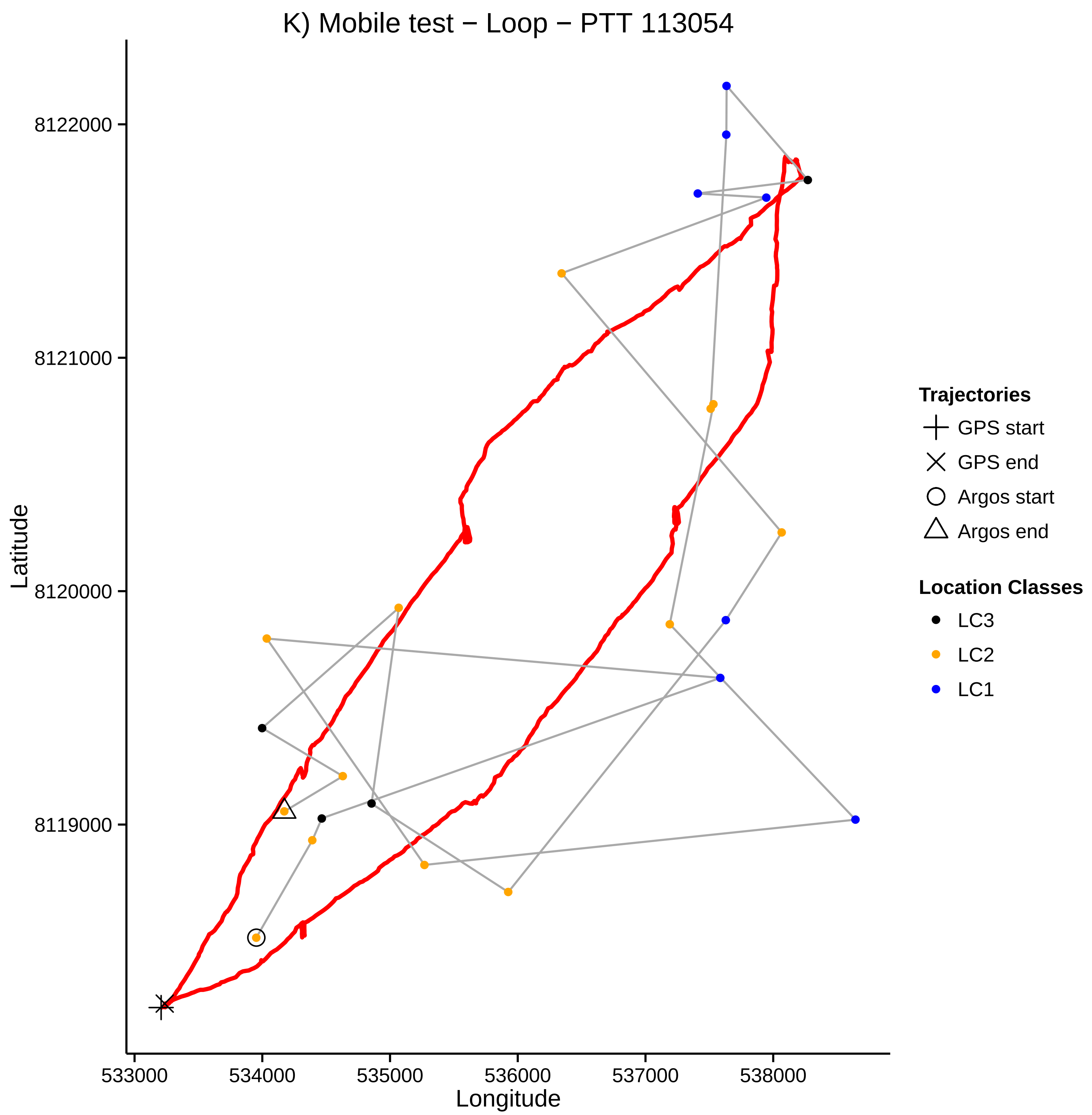

K) Mobile test − Loop − PTT 113054
Latitude
8122000
8121000
8120000
8119000
Longitude
533000
534000
535000
536000
537000
538000
Trajectories
GPS start
GPS end
Argos start
Argos end
Location Classes
LC3
LC2
LC1

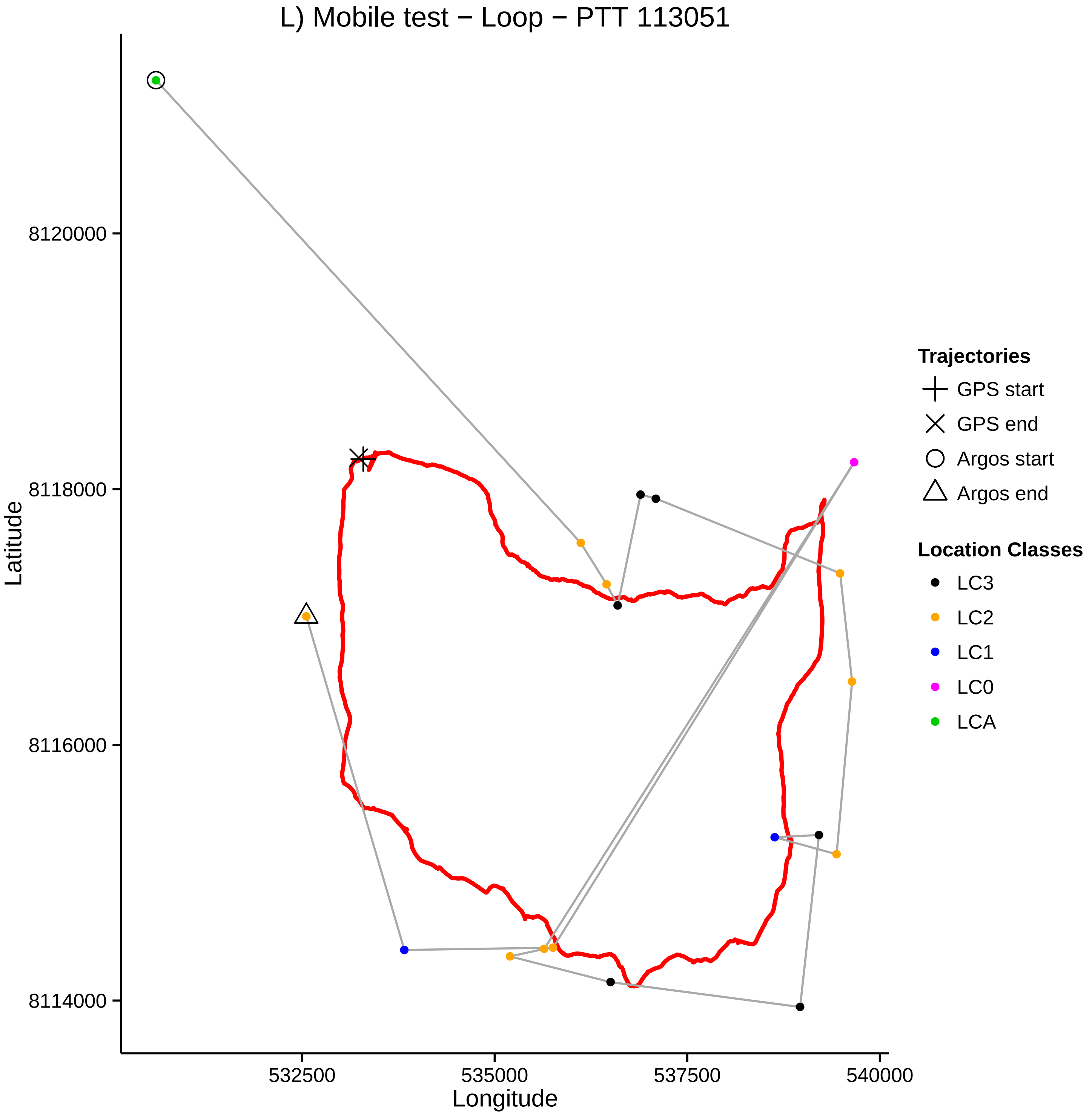

L) Mobile test − Loop − PTT 113051
Latitude
Longitude
8120000
8118000
8116000
8114000
532500
535000
537500
540000
Trajectories
GPS start
GPS end
Argos start
Argos end
Location Classes
LC3
LC2
LC1
LC0
LCA
