# Supplementary material for: Evaluation of Argos Telemetry Accuracy in the High-Arctic and Implications for the Estimation of Home-Range Size
Source: PLoS One. 2015 Nov 6;10(11):e0141999. doi: 10.1371/journal.pone.0141999 (PMC4636246; doi:10.1371/journal.pone.0141999)
Supplement: S1 Table — (DOCX) [file pone.0141999.s004.docx]

**Table S1 :** Differences of least squared means between the fixed factors of mixed models.

|  |  | **Size ratio** | | **Proportion in reference** | |
| --- | --- | --- | --- | --- | --- |
|  |  | **MCP** | **Kernel** | **MCP** | **Kernel** |
| **Observed** | Raw - LC32 | **5.62 (<0.001)** | **7.57 (<0.001)** | **-1.15 (0.25)** | **-6.09 (<0.001)** |
|  | Raw - LC321 | **4.71 (<0.001)** | **5.03 (<0.001)** | **-1.06 (0.29)** | **-3.65 (<0.001)** |
|  | Raw - DAF | **5.65 (<0.001)** | **6.98 (<0.001)** | **-0.96 (0.34)** | **-5.08 (<0.001)** |
|  | Raw - HSF | **5.77 (<0.001)** | **6.93 (<0.001)** | **-1.89 (0.06)** | **-5.27 (<0.001)** |
|  | LC32 - LC321 | -0.91 (0.4) | **-2.55 (0.01)** | 0.09 (0.93) | **2.44 (0.02)** |
|  | LC32 - DAF | 0.03 (1) | -0.6 (0.55) | 0.18 (0.85) | 1 (0.32) |
|  | LC32 - HSF | 0.15 (0.9) | -0.64 (0.52) | -0.74 (0.46) | 0.81 (0.42) |
|  | LC321 - DAF | 0.94 (0.4) | 1.95 (0.06) | 0.09 (0.93) | -1.44 (0.16) |
|  | LC321 - HSF | 1.06 (0.3) | 1.9 (0.06) | -0.84 (0.41) | -1.62 (0.11) |
|  | DAF - HSF | 0.12 (0.9) | -0.05 (0.96) | -0.93 (0.36) | -0.19 (0.85) |
| **Simulated** | LC32 - Static | **-2.25 (0.03)** | **-4.91 (<0.001)** | **6.69 (<0.001)** | **2.37 (0.03)** |
|  | LC32 - Mobile | -1.6 (0.13) | -1.58 (0.12) | **3.32 (0.004)** | 1.65 (0.12) |
|  | Static - Mobile | **3.34 (0.003)** | **19.23 (<0.001)** | **-12.02 (<0.001)** | **-36.7 (<0.001)** |

Home-range size estimations were conducted using 95% MCP and 95% kernel (h = 850, cell grid size = 250 m). The *t*-values of the differences and the *P*-values are between parentheses. Significative differences (p < 0.05) are shown in bold.
